# Serosurvey of *Coxiella burnetii* in Descendants of Former Black Slaves (Quilombola Communities) of Southern Brazil

**DOI:** 10.3390/microorganisms12010092

**Published:** 2024-01-02

**Authors:** Danilo Alves de França, Louise Bach Kmetiuk, Giovanni Augusto Kalempa Panazzolo, Orlei José Domingues, Filipe Pereira da Silva, Leandro Meneguelli Biondo, Mateus de Souza Ribeiro Mioni, Fábio Sossai Possebon, Ana Íris de Lima Duré, Marcos Vinicius Ferreira Silva, Myrian Morato Duarte, Giovani Marino Fávero, Alexander Welker Biondo, Helio Langoni

**Affiliations:** 1 Department of Animal Production and Preventive Veterinary Medicine, School of Veterinary Medicine and Animals Science, São Paulo State University, Botucatu 18618-681, SP, Brazil; danilo.franca@unesp.br (D.A.d.F.); fabio.possebon@unesp.br (F.S.P.); 2 Department of Veterinary Medicine, Federal University of Paraná State, Curitiba 80035-050, PR, Brazil; lkmetiuk@sms.curitiba.pr.gov.br (L.B.K.); abiondo@ufpr.br (A.W.B.); 3 Graduate College of Pharmaceutical Sciences, State University of Ponta Grossa, Ponta Grossa 84030-900, PR, Brazil; giovanni.panazzolo@uepg.br (G.A.K.P.); ojdsoares@uepg.br (O.J.D.); gmfavero@uepg.br (G.M.F.); 4 Service of Virology and Rickettsiosis, Octavio Magalhaes Institute, Ezequiel Dias Foundation, Belo Horizonte 30510-010, MG, Brazil; filipeps.1919@gmail.com (F.P.d.S.); ana.dure@funed.mg.gov.br (A.Í.d.L.D.); marcos.silva@funed.mg.gov.br (M.V.F.S.); myrian.duarte@funed.mg.gov.br (M.M.D.); 5 National Institute of the Atlantic Forest (INMA), Brazilian Ministry of Science, Technology and Innovation, Santa Teresa 29650-000, ES, Brazil; leandro.biondo@inma.gov.br; 6 Interdisciplinary Graduate Studies, University of British Columbia, Kelowna, BC V1V 1V7, Canada; 7 Department of Pathology, Reproduction and One Health, Faculty of Agricultural and Veterinary Sciences, São Paulo State University, Jaboticabal 14884-900, SP, Brazil; mateus.mioni@unesp.br

**Keywords:** Q fever, vulnerable populations, seroprevalence, indirect immunofluorescence assay

## Abstract

Brazilian descendants of former Black-slave (quilombola) communities have been predisposed to several zoonotic diseases due to social vulnerability, characterized by subsistence and close contact with livestock and companion animals. Accordingly, the present study has assessed anti-*Coxiella burnetii* antibodies in 200 individuals and 20 dogs from four quilombola communities located in Paraná State, southern Brazil. Serum samples were tested by indirect immunofluorescence assay (IFA) using in-house and commercial diagnostic protocols, with analysis of seropositive titers and antibody type. Fisher’s exact test was used to compare seropositivity to *C. burnetti* with binary variables, with variables with three or more possible responses submitted to logistic regression. In total, 44/200 (22%; 95% CI 16.82–28.24) people tested positive, and 4.5% had titers higher than 128, indicating a recent onset of *C. burnetii* infection. Seropositive individuals were statistically associated with the Limitão community (*p* = 0.0013), urban workers as occupations (*p* = 0.0475), consumption of undercooked meat (*p* = 0.0159), and contact with animal abortion (*p* = 0.0276). No seropositivity association was found for age, sex, education, habit of entering forest areas, consumption of game meat, consumption of raw milk, flea and tick bites, dog contact, or history of female miscarriage. Only one of 20 dogs was seropositive with a titer of 128, probably related to an acute animal infection. Despite the prevalence here being higher than previous Brazilian reports, including with symptomatic populations, the results were within range for worldwide outbreaks and occupational risk populations. To the reader’s knowledge, this is the first human survey of Q fever in southern Brazil and should be considered a warning for *C. burnetii* in vulnerable populations, particularly Quilombola communities.

## 1. Introduction

Despite the fact that *Coxiella burnetii*, the causative agent of Q fever, has been mostly described as an asymptomatic infection, over time manifestation may lead to an acute self-limiting disease or a persistent focalized infection with fatal consequences [1]. Due to the bacterial ability to persist within the cell phagolysosomes for years, *C. burnetii* has been frequently reported in cardiac patients and with vascular infections [2,3,4,5,6]. Pathogen transmission may include contact with infected animals, inhalation of contaminated aerosols, and, to a lesser extent, consumption of contaminated raw milk, tick bites, and fomites [7]. The infection outcome depends on the individual susceptibility and the transmission route, resulting in a lower or higher infective dose [7].

**Figure 1:**
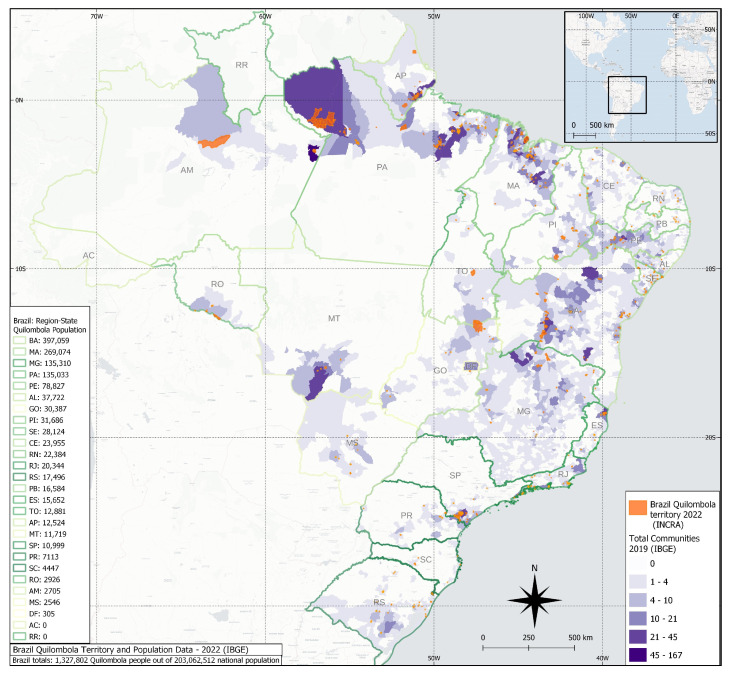
Map of current quilombola communities in Brazil, with state distribution per population and density (purple), with the recent areas officially recognized in 2022 (orange) by INCRA (Brazilian Government).

Brazil has been the home of the largest African-descendant population outside Africa, with a history of slavery resistance and establishing rural communities called “quilombos,” distributed mainly in remote and isolated areas, maintaining their ancestral culture, and living a life characterized by subsistence agriculture and livestock raising [8]. The latest Brazilian 2022 census has shown an overall population of 1.3 million quilombo individuals (0.65% of the total population), distributed in 1696/5568 (30.45%) Brazilian cities, with the highest population living in the northeastern (905,415; 68.2%) and the lowest in the southern region (29,056; 2.19%) (Figure 1) [9].

Human infection has been associated with direct contact with domestic ruminants and living in rural areas, which may contribute to *C. burnetti* spreading [10]. Although quilombola individuals and their dogs may be exposed to *C. burnetti*, no study to date has assessed these Brazilian quilombola populations. Accordingly, the aim of the present study was to assess the presence of anti-*Coxiella burnetii* antibodies and associated risk factors in humans and dogs from quilombola communities in southern Brazil.

## 2. Materials and Methods

### 2.1. Study Design and Sample Collection

This study was conducted with humans (*n* = 200) and dogs (*n* = 20) belonging to four Brazilian quilombola communities located in rural regions of the state of Paraná, southern Brazil. The four quilombos (Limitão, Mamans, Serra do Apon, and Tronco) were established during the late 19th century by individuals fleeing and seeking refuge from Black slavery. Livelihoods primarily cultivated yerba mate plants and engaged in livestock-related activities, later through subsistence agriculture, rudimentary livestock, and handmade crafting [11]. These communities were located about 50 to 60 km (31 to 37 miles) from the main roads, solely connected by unmaintained gravel and sand roads at the time [11]. 

Samplings in this Q fever study were obtained by convenience and were part of a public policy project conducted by the same on-field group for COVID-19 screening, not in the context of comparing communities but rather targeting specific vulnerable populations in partnership with public healthcare institutions during pandemics. The quilombola population of Paraná was recently estimated at 7113 individuals, officially recognized as direct descendants of blacks from colonial times [9]. A representative sample size was proportionally calculated using open-source epidemiological statistics (Openepi) [12]. The calculation was made considering a regional population of 4000 quilombola individuals with a precision of 5%, a confidence interval of 95%, and a frequency of 12.68%, considering the average prevalence obtained in studies in nearby states [10].

The on-field samplings were performed in a series of six incursions from December 2021 to March 2022, preceded by door-to-door visits organized locally by community leaders and city healthcare workers. The four quilombola communities herein were part of the Rural Association of Castro County Quilombola Communities, established in 2005 for empowering member communities, preserving their African heritage, and advocating for public policies. The research taskforce team comprised certified nurses, pharmacists, veterinarians, and biologists, visiting the quilombola communities in a convoy of equipped vehicles with sampling tables, chairs, field tents, and blood sampling materials. As an immediate health approach, all dogs brought for sampling were vaccinated, dewormed, and given anti-flea drugs. All dogs have shown the presence of ticks and/or fleas at the moment of blood sampling. This study area included both natural and anthropized regions of the Atlantic Forest and Cerrado biomes, under a moist temperate climate with 17.5 °C of annual average temperature and 1495 mm^3^ of average precipitation. All individuals voluntarily participated, were sampled after signing a formal consent, and responded to an epidemiological questionnaire. A total of 10 mL of blood samples were collected from humans by cephalic puncture, performed by certified nurses, and from dogs by jugular venipuncture, after physical restraint and performed by certified veterinarians. All samples were collected in tubes without anticoagulant and centrifuged at 1500 revolutions per minute for five minutes, serum separated, and stored at −80 °C until processing.

### 2.2. Human Serological Testing

The human serum samples were tested by indirect immunofluorescence assay (IFA), performed at the Laboratory of Rickettsioses and Hantaviruses of the Octávio Magalhães Institute, Ezequiel Dias Foundation (Funed-MG). A previously validated in-house assay (IFA Kit, Belo Horizonte, Brazil) was used with an antigen produced from embryonated eggs of the Argentine strain At12 isolated from ticks [13] and tested using an anti-human IgG conjugate antibody (Bethyl Laboratories, Montgomery, TX, EUA). Positive and negative controls were obtained from a previously laboratory daily routine, with positive titers of 1:16.

In short, serum aliquots at a dilution of 1:16 in phosphate buffered saline (PBS, 0.1 M, pH 7.2) were placed on antigen-containing (30 µL) slides, incubated (37 °C for 30 min), washed with PBS, and then placed in a humidity chamber to dry. Following, 30 µL of fluorescein isothiocyanate (FITC)-anti-human IgG antibody was inserted into the concavities, with another incubation in a humid chamber for 30 min at 37 °C. After another washing and drying, slides were mounted with buffered glycerin and coverslips with immunofluorescence readings under a specific microscope (Olympus BX53, Tokyo, Japan) with a 40x optical objective. Positive and negative reaction controls were prepared for each slide reading, with positive samples submitted to serial dilutions of 1:16, 1:32, 1:64, and so on until the last titer was reached, as previously described [13].

### 2.3. Dog Serological Testing

Dog serum samples were submitted to the same protocol of indirect immunofluorescence assay (IFA) used in human samples. In addition, a commercially available kit (SCIMEDX Corporation, Denville, NJ, USA) containing phase I and phase II antigens of *Coxiella burnetii* Nine Mile was used. The procedure was the same as that used in human testing, except for the use of a fluorescein isothiocyanate (FITC) anti-dog antibody (Zoonosis Control Center, São Paulo, Brazil).

### 2.4. Data Analysis

Fisher’s exact test was used to compare the prevalence of *C. burnetii* seropositivity for the binary variables. The statistical level was considered significant at *p* ≤ 0.05. The analyses were performed using Statistical Analysis Software Studio 3.81 (SAS) (SAS Institute Inc., Cary, NC, USA).

## 3. Results 

**Figure 2:**
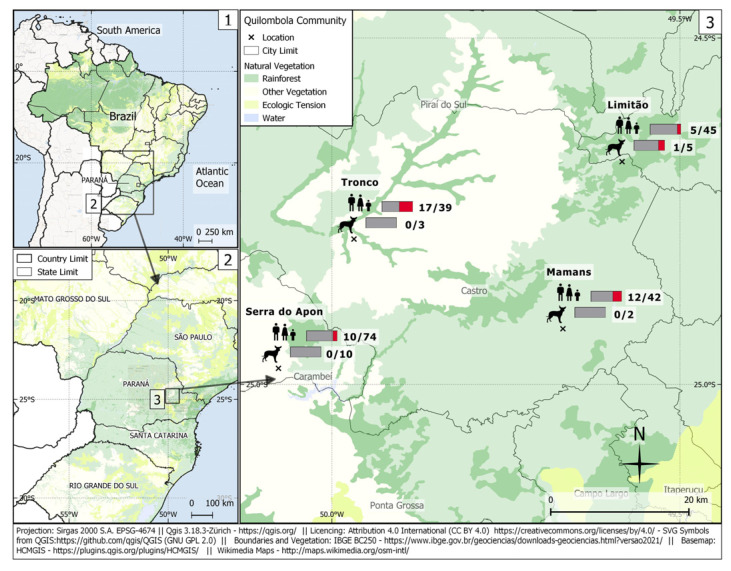
**1**. Map of Brazil, with closeup location of Paraná state. **2**. Map of Paraná State, with a closeup of the region in which this study was performed. **3**. Map of Castro municipality, with the distribution of seropositive cases of anti-*Coxiella burnetii* antibodies in the human and dog quilombola populations studied. Accessed on: 20 January 2023.

A total of 44/200 (22.0%; 95% CI 16.82–28.24) human samples were seropositive for *C. burnetii*. The titers of positive samples varied from 1:16 in 14/200 (7.0%), 1:64 in 21/200 (10.5%), and 1:128 or higher in 9/200 (4.5%) individuals. Overall, seropositivity was observed in only one/20 dogs from the Limitão community, with a 1:128 titer for the phase II antibody, suggesting an acute infection (Figure 2). This seropositive dog was 11 years old, male, clinically healthy, of mixed breed, and used to eating raw meat. This dog frequented forest areas and did not receive anti-parasite prophylaxis.

Seropositivity to *C. burnetti* was statistically associated with farm workers’ occupation (*p* = 0.0475), consumption of undercooked meat (*p* = 0.0159), and contact with animal abortion (*p* = 0.0276) (Table 1). No association was found considering age (*p* = 0.5035), sex (0.7755), education level (*p* = 0.3826), access to forest areas (*p* = 0.4820), consumption of game meat (*p* = 0.0761), consumption of raw milk (*p* = 0.3513), flea bites (*p* = 0.5250), tick bites (*p* = 0.5768), history of miscarriage (*p* = 0.3529), and dog breeding (0.2033).

Seropositive quilombola individuals were more likely to consume rare meat to consume undercooked (50.0%) than well-cooked (19.9%) meat, to have contact with animal abortion and fomites (36.8%) than not (18.5%), and to work on farms (32.7%) than in urban areas (18.5%). Out of the titers ≥ 1:128, 5/9 (55.6%) persons lived in the Tronco community, 3/7 (42.9%) consumed undercooked meat, and 3/9 (33.3%) had contact with animal abortion.

## 4. Discussion 

The human seropositivity of 44/200 (22.0%) individuals herein has been the second highest reported to date in Brazil, surpassed only by a series of reported Q fever cases with 10/16 (62.5%) in Minas Gerais state, Brazil [14]. All previous Brazilian serosurveys have reported lower seropositivity, including 1/61 (1.6%) patients with infective endocarditis in Sao Paulo state [15], 4/125 (3.2%) HIV-positive patients in Rio de Janeiro state [16], 17/437 (3.9%) in healthy individuals from Minas Gerais state, southeastern [17], 25/437 (5.72%) Dengue suspected patients in Minas Gerais state [18], 4/51 (7.8%) patients with culture-negative endocarditis in São Paulo state [19], 26/272 (10.0%) Dengue suspected patients in Rio de Janeiro state [20], and 129/604 (21.4%) Dengue suspected patients in São Paulo state [21]. If considering the Tronco community itself, with 17/39 (43.6%) seropositive individuals, the study herein had the highest reported frequency in healthy populations in Brazil.

Despite these few studies, Brazil still has the highest number of Q fever surveys in Latin America, followed by French Guyana, Colombia, Ecuador, and Argentina [22]. French Guiana presented an incidence rate (100,000 inhabitants/year) of 37 cases from 1990 to 2006, with 132 sampled patients [23], and of 27.4 cases from 2007 to 2017, with 695 sampled patients [24]. In addition, French Guiana reported Q fever in 32/131 (24.4%) patients with pneumonia and 25/275 (9.1%) with suspected Dengue [25,26]. A total of 83/153 (54.2%) cases were reported in slaughterhouse workers in Medellín, Colombia [27], 13/54 (24.1%) in veterinary professionals and students in Quito, Ecuador [28], and 1/99 (1.0%) in the healthy population of Buenos Aires, Argentina [29]. The study of vulnerable populations has brought a different perspective on how zoonotic diseases affect populations, alerting them to new associated risk factors and demanding a One Health approach to human, animal, and environmental health [30]. In the Brazilian Amazon, an indigenous population was investigated, which resulted in no (0/73) seropositive individuals [31]. In French Guyana, a cross-sectional study of illegal miners showed 11/380 (2.9%) seropositivity [32]. Based on such findings, the quilombola seropositivity (22.0%) herein becomes the highest in Latin America to date, highlighting the exposure of vulnerable Brazilian populations.

Although this is the highest Latin American prevalence in a healthy population, a study in Africa showed 86/137 (62.8%) seropositive pastoralists in Nigeria, likely due to livestock contact on rural farms [33]. Not surprisingly, large epidemics have occurred in farm populations in the Netherlands and Australia. Pathogen maintenance in such populations may become a serious human and animal health issue, impairing disease control and leading to social and economic losses [34,35].

Q fever has been indicated as an underestimated disease and an emerging health concern in Brazil [10]. Molecular and serological surveys have found, respectively, 44/360 (12.2%) positive bovines [36] and 129/604 (21.4%) positive humans [21] in São Paulo state, southeastern Brazil. The occurrence of Q fever has been evidenced since 2016 in Rio de Janeiro state [20,37].

The serological 1:128 cutoff point has been defined to determine acute disease in persons with Q fever [38]. Although the study herein has been designed for a survey of four clinically healthy quilombola populations, 9/200 (4.5%) of the total, or 9/44 (20.4%) of positive individuals, presented ≥1:128 serology, suggesting a recent infection. No titer ≥1:800 was found, considered a chronic disease characteristic [38]. Noteworthy, *C. burnetii* can remain in the body for years, even in asymptomatic cases, later developing serious complications such as endocarditis and hepatitis [4,5,39].

In the present study, we noted that individuals living in quilombola communities showed significant seropositivity for *C. burnetii* as a reflection of the sum of rural habits and characteristics, such as proximity to cattle. When eliminated by aerosols at the time of animal calving or as a result of abortions, *C. burnetii* spores attach themselves to dust and travel by wind, reaching up to 30 km from the aerosolization site [40]. Similarly, workers who spend most of the day on farms were more seropositive than those working in urban environments, probably due to their contact with cattle. 

Consumption of undercooked meat was associated with *C. burnetii* seropositivity. Although the role of raw and undercooked meat in *C. burnetii* remains to be described, molecular studies have detected *C. burnetii* in meat samples of cattle, buffalo, goats, and sheep collected from the slaughterhouses of Pakistan [41] and in raw meat destined exclusively for companion animal consumption in Australia [42].

The epidemiology of Q fever remains to be clarified, demanding an investigation of all possible associated risk factors. Although milk contamination by *C. burnetti* has been reportedly observed [40,43,44], its presence was not statistically associated with population seropositivity, and raw milk consumption remains to be fully established as an important human transmission route. On the other hand, the consumption of undercooked meat was significantly associated with seropositivity. Contact with ectoparasites does not seem to be an important factor for human infection, although ticks may be commonly infected [45,46]. Despite contact with dogs not being a risk factor, contact with dog abortions and vaginal fluids has been significant for infection [45]. Although not statistically significant, abortion in women was highly referred to and may require more attention, since 5/16 (31.3%) of the women who mentioned abortion were seropositive. Data on human abortions resulting from Q fever has been scarce, and such a risk factor should be further investigated.

Dogs may become at risk of infection at the time of birth or in cases of abortion [47]. In other situations, dogs may not present a risk of transmission to humans or other animals [10]. The existence of only one seropositive dog in the present study and in the community with a lower percentage of seropositive human samples may indicate that dogs have distinct infection routes. Shedding patterns of *C. burnetii* in dogs have not been assessed, and the association between dogs and human infection is a query to be evaluated. Nevertheless, the serological outcome of our survey may suggest that dogs are not a potential source of *C. burnetii* for humans. Although there was low dog sampling, this is the first study that concomitantly accessed human and dog populations under socioeconomic vulnerability. In the present study, human seropositivity was associated with canine abortion contact. Thus, further studies should be conducted to clarify the role of dogs in *C. burnetti* transmission in such areas.

Despite small domestic ruminants being recognized as its main transmission source [48], *C. burnetii* was also reported in large ruminants and detected in bovine abortion remains in southeastern Brazil [36]. There were approximately 213,000 cattle farms in Paraná State at the time of the survey, with approximately 6.3 million cattle heads and dairy production accounting for 25% of the local economy [49]. Although *C. burnetii* in wildlife has been studied worldwide [48,50,51], few studies were conducted in Brazil, including detection in 6/131 (4.6%) of the wild rodents of southeastern Brazil [52], in 4/21 (19.0%) *Artibeus* spp. bats in northeastern Brazil [53], and in 9/169 (5.32%) deer in southeast and central-western Brazil [54]. In addition, future studies should also investigate the role of other livestock populations in *C. burnetti* transmission in such communities in Paraná State.

Our study presents some limitations regarding its results. First, we have not performed IgM detection. Although IgM antibodies may provide ancillary information to IgG titers in human serodiagnosis, IgM specificity has been much lower than IgG, with higher cross-reactivity, making it not ideally the best *C. burnetii* detection tool in exposed populations [38,55]. In addition, an in-house serological test for Q fever was used instead of commercially available kits, which may have generated differences in positivity and correspondent titers. However, the in-house assay produced for the study herein with the Argentine antigen has proven to be very efficient in detecting anti-phase II antibodies, i.e., IgG anti-*C. burnetii* antibodies, in patients with Q fever [13].

As this study was a convenience survey with no random sampling, biased outcomes may be a limitation, as statistical differences on associated risk factors among groups may not represent the actual possibilities. However, as Q fever epidemiology has been poorly understood, the inferences herein may still be valuable as potential insights and associated causes of exposure in quilombola populations. Bearing in mind such limitations, the data herein have indicated the habits of communities as potential associated risk factors, which may also impact other infectious diseases and therefore common prevention measures.

Despite the fact that the health impact of *C. burnetii* on the studied population could not be confirmed, as a healthy population was tested in a single sampling, the primary aim herein was to assess the *C. burnetti* exposure in this vulnerable population. As *C. burnetti* was detected in the quilombola population by sole sampling, further studies should screen and investigate febrile cases in quilombola communities locally, as fever may reappear further, being reactivated in seropositive individuals. Nonetheless, despite not characterizing active infections or differentiating acute from persistent infection, the evidence herein of anti-*C. burnetti* antibodies has indicated exposure in such a vulnerable population and should be further investigated.

## 5. Conclusions

To the author’s knowledge, this is the first survey of human and animal populations for *C. burnetti* in southern Brazil. This study has reported high *C. burnetii* seropositivity in the quilombola population, likely associated with close contact with livestock. Such results may provide important findings for future public health actions to prevent *C. burnetii* infection in overlapping areas of vulnerable human populations, livestock, and wildlife.

## Figures and Tables

**Table 1:** Sociodemographic characteristics of the quilombola population in the state of Paraná, Brazil (2023) and their respective seropositivity for Q fever.

Variables	*C. burnetii* Seronegative		*C. burnetii* Seropositive		Total Population
	N	%	N	%	N
Quilombola community					
Limitão	40	88.9	5	11.1	45
Mamans	30	71.4	12	28.6	42
Serra do Apon	64	86.5	10	13.5	74
Tronco	22	56.4	17	43.6	39
Age					
Young (1 to 18)	36	81.8	7	18.2	44
Adults (19 to 59)	96	75.6	31	24.4	127
Elderly (≥60)	24	82.8	5	17.2	29
*p* value = 0.5035					
Sex					
Female	92	78.0	26	22.0	118
Male	65	79.3	17	20.7	82
*p* value = 0.7755					
Education					
Illiteracy	29	80.6	7	19.4	36
Elementary school	103	77.4	30	22.6	133
High school	21	84.0	4	16.0	25
Graduate	3	50.0	3	50.0	6
*p* value = 0.3826					
Occupation					
Rural Workers	33	67.3	16	32.7	49
Urban Workers	123	82.5	28	18.5	151
*p* value = 0.0475					
Access forest areas					
Yes	134	78.8	36	21.2	170
No	24	80.0	6	20.0	30
*p* value = 0.4820					
Game meat consumption					
Yes	23	92.0	2	8.0	25
No	134	76.6	41	23.4	175
*p* value = 0.0761					
Meat consumption					
Undercooked	7	50.0	7	50.0	14
Well-done	149	80.1	37	19.9	186
*p* value = 0.0159					
Raw milk consumption					
Yes	114	79.7	29	20.3	143
No	42	73.7	15	26.3	57
*p* value = 0.3513					
Flea bites					
Yes	127	78.9	34	21.1	161
No	29	74.4	10	25.6	39
*p* value = 0.5250					
Tick bites					
Yes	111	79.3	29	20.7	140
No	45	75.0	15	25.0	60
*p* value = 0.5768					
Miscarriage					
Yes	11	68.7	5	31.3	16
No	81	79.4	21	20.6	102
*p* value = 0.3529					
Dog breeding					
Yes	141	79.7	36	20.3	177
No	14	60.9	8	34.8	23
*p* value = 0.2033					
Animal abortion contact					
Yes	24	63.2	14	36.8	38
No	132	81.5	30	18.5	162
*p* value = 0.0276					

*p* values in bold indicate significant differences (*p* < 0.05) within the categories.

## Data Availability

All relevant data are within the manuscript.
